# Assessing the role of tumour-associated macrophage subsets in breast cancer subtypes using digital image analysis

**DOI:** 10.1007/s10549-022-06859-y

**Published:** 2023-01-09

**Authors:** Mieke C. Zwager, Rico Bense, Stijn Waaijer, Si-Qi Qiu, Hetty Timmer-Bosscha, Elisabeth G. E. de Vries, Carolien P. Schröder, Bert van der Vegt

**Affiliations:** 1 Department of Pathology and Medical Biology, University of Groningen, University Medical Center Groningen, Groningen, The Netherlands; 2 Department of Medical Oncology, University of Groningen, University Medical Center Groningen, Groningen, The Netherlands; 3 Diagnosis and Treatment Center of Breast Diseases, Clinical Research Center, Shantou Central Hospital, Shantou, China; 4 Guangdong Provincial Key Laboratory for Breast Cancer Diagnosis and Treatment, Shantou University Medical College, Shantou, China; 5 Department of Pathology and Medical Biology, University of Groningen, University Medical Center Groningen, PO Box 30001, 9700 RB Groningen, The Netherlands; 6 Department of Medical Oncology, Dutch Cancer Institute, Amsterdam, Netherlands

**Keywords:** Breast cancer, Tumour-associated macrophage (TAM), Immunohistochemistry (IHC), Digital image analysis (DIA), CD68, CD163, CSF-1R, CD206

## Abstract

**Purpose:**

The number of M1-like and M2-like tumour-associated macrophages (TAMs) and their ratio can play a role in breast cancer development and progression. Early clinical trials using macrophage targeting compounds are currently ongoing. However, the most optimal detection method of M1-like and M2-like macrophage subsets and their clinical relevance in breast cancer is still unclear. We aimed to optimize the assessment of TAM subsets in different breast cancer subtypes, and therefore related TAM subset numbers and ratio to clinicopathological characteristics and clinical outcome.

**Methods:**

Tissue microarrays of 347 consecutive primary Luminal-A, Luminal-B, HER2-positive and triple-negative tumours of patients with early-stage breast cancer were serially sectioned and immunohistochemically stained for the pan-macrophage marker CD68 and the M2-like macrophage markers CD163, CSF-1R and CD206. TAM numbers were quantified using a digital image analysis algorithm. M1-like macrophage numbers were calculated by subtracting M2-like TAM numbers from the total TAM number.

**Results:**

M2-like markers CD163 and CSF-1R showed a moderate positive association with each other and with CD68 (*r* ≥ 0.47), but only weakly with CD206 (*r* ≤ 0.06). CD68 + , CD163 + and CSF-1R + macrophages correlated with tumour grade in Luminal-B tumours (*P* < 0.001). Total or subset TAM numbers did not correlate with disease outcome in any breast cancer subtype.

**Conclusion:**

In conclusion, macrophages and their subsets can be detected by means of a panel of TAM markers and are related to unfavourable clinicopathological characteristics in Luminal-B breast cancer. However, their impact on outcome remains unclear. Preferably, this should be determined in prospective series.

**Supplementary Information:**

The online version contains supplementary material available at 10.1007/s10549-022-06859-y.

## Introduction

Breast cancer is the most common malignancy and the leading cause of cancer-related death in women worldwide [1, 2]. Despite early detection and improved treatment, breast cancer still accounts for 15% of cancer-related deaths [3]. Treatment effects differ between patients and breast tumours are known to become therapy-resistant, necessitating new treatment modalities [4, 5].

Increasingly, it is becoming clear that tumour-associated macrophages (TAMs) in the tumour microenvironment are involved in breast cancer development, progression and therapy response [6–8]. Therefore, they may become a target for therapy [9–11]. Macrophages can be characterized as classically activated anti-tumour M1-like macrophages and alternatively activated pro-tumour M2-like macrophages [12, 13]. TAMs in the breast carcinoma microenvironment predominantly display the M2-like phenotype. Preclinically, they promote tumour growth, invasion, metastasis, angiogenesis and therapy resistance [14–21].

In an in silico analysis of publicly available gene expression profiles of 7270 primary tumours of patients with non-metastatic breast cancer (prior to any treatment), we previously found that a higher fraction of M0 macrophages was associated with shorter disease-free survival (DFS) and overall survival (OS) in oestrogen receptor (ER)-positive disease, while a higher fraction of M1 macrophages was associated with a higher pathological complete response rate and prolonged OS [22]. Assessing the ratio between TAM subset numbers in breast cancer subtypes is therefore likely of importance.

Unfortunately, TAM subsets’ most optimal immunohistochemical detection method is unknown, and the clinical implications of the immunohistochemically defined subsets are unclear. Moreover, most studies to date do not distinguish between M1-like and M2-like TAMs, and studies assessing their ratio or comparing the multiple M2-like macrophage markers in breast cancer subtypes are lacking [23, 24].

Manual counting of TAM subsets is labour intensive and prone to inter- and intra-observer variability. Digital image analysis (DIA) is an efficient method for quantifying macrophages and other immune cell types in breast cancer [25]. DIA may therefore aid standardized, objective quantitative TAM assessment.

In light of the above, we aimed to optimize the assessment of TAM subsets in breast cancer subtypes. Therefore, we related TAM subset numbers and ratio to clinicopathological characteristics and clinical outcome. We used DIA to quantify CD68 (pan-macrophage marker), CD163 (M2-like TAM marker), CSF-1R (colony-stimulating factor 1 receptor; M2-like TAM marker) and CD206 (M2-like TAM marker) positive cells in a large, well-characterized series of Luminal-A, Luminal-B, human epidermal growth factor receptor 2 (HER2)-positive and triple-negative breast cancers (TNBC).

## Materials and methods

### Patients

**Fig. 1:**
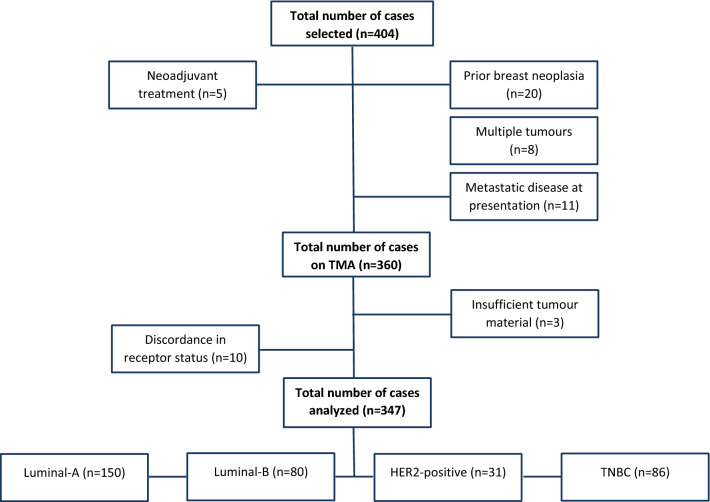
CONSORT flow diagram describing sample selection. After initial patient selection, 44 cases were excluded. After histological and immunohistochemical evaluation of the TMA sections, 13 cases were excluded

Consecutive resection specimens of HER2-positive, triple-negative and the first 200 ER-positive/HER2-negative primary, non-metastasized, breast carcinomas diagnosed in the University Medical Center Groningen (The Netherlands) between 2006 and 2017 were retrospectively collected. Samples of 57 patients were excluded, resulting in a study population of 347 patients with primary invasive breast carcinoma (Fig. 1). All tumours were reviewed for diagnosis and tumour grade on diagnostic haematoxylin and eosin (H&E)-stained slides by two of the authors (MZ and BvdV). Retrospective collection of clinicopathological characteristics and overall survival data from patient charts and the Personal Records Database was approved by the Local Ethics Review Board Pathology non-WMO studies (UMCG research register number 201900243, approved on 18-8-2020) and according to UMCG security guidelines, in line with Dutch law. Information was retrieved on age, treatment regimen, tumour size, lymph node status, lymphovascular invasion, DFS and OS. DFS was defined as the interval between date of diagnosis and date of local recurrence, regional recurrence, distant metastasis, second primary breast cancer or death by any cause. OS was defined as the interval between date of diagnosis and date of death by any cause.

The specimens used in this study were obtained from redundant diagnostic material stored at the Department of Pathology. No objection to research on redundant tissue was recorded from these patients in the institutional record of objection.

### Tissue microarray

Tissue microarrays (TMAs) were assembled using the formalin-fixed, paraffin-embedded tumour blocks. Three representative 0.6 mm tumour cores of each donor block were transferred into recipient paraffin blocks using a Manual Tissue Arrayer (Beecher Instruments, WI, USA) to account for tumour heterogeneity. In total, seven TMAs were constructed, each containing tumour samples from 42–71 patients and healthy control tissue samples. Serial sections of 3 μm were cut with a standard microtome.

### Immunohistochemistry

Immunohistochemistry of ER (SP-1, Ventana), progesterone receptor (PR) (1E2, Ventana), HER2 (SP-3, Thermo Fisher Scientific), Ki67 (30–9, Ventana), CD68 (KP-1, Roche), CD163 (MRQ26, Ventana), CSF-1R (5c11, Sigma-Aldrich) and CD206 (SP211, Sigma-Aldrich) was performed. For ER, PR, HER2, Ki67, CD68 and CD163, antibodies were pre-diluted by the manufacturer and sections were stained on a Ventana Benchmark Ultra immunostainer (Ventana) according to the manufacturer’s protocols.

For CSF-1R and CD206, the following immunostaining protocol was performed: sections were deparaffinized, rehydrated in a series of decreasing concentrations of alcohol and washed with demineralized water. Antigen retrieval was performed by cooking the sections in the microwave for 15 min in 10 mM citrate buffer (pH 6.0). Endogenous peroxidase reaction was blocked by incubating the sections in 0.3% H_2_O_2_ in 50 ml phosphate buffered saline (PBS) [0.15 M NaCl, 8.0 mM Na_2_HPO_4_ 2 H_2_O, 1.5 mM KH_2_PO_4_], (pH 7.4)] for 30 min.

The primary antibodies were diluted (1:200) in PBS containing 1% bovine serum albumin (BSA) and incubated for 1 h. The secondary antibodies for CSF-1R (polyclonal goat anti-rabbit [GAR^PO^], DAKO, 1:100 diluted in PBS containing 1% BSA and 1% AB-serum) and CD206 (polyclonal rabbit anti-mouse [RAM^PO^], DAKO, 1:100 diluted in PBS containing 1% BSA and 1% AB-serum) were incubated for 30 min, after which the tertiary antibodies for CSF-1R (polyclonal rabbit anti-goat [RAG^PO^], DAKO, 1:100 diluted in PBS containing 1% BSA and 1% AB-serum) and CD206 (GAR^PO^) were incubated for 30 min. Visualization was performed using the diaminobenzidine peroxidase reaction. Sections were counterstained with haematoxylin and dehydrated in a series of increasing concentrations of alcohol.

### Evaluation of immunohistochemistry

Scoring of ER and PR was based on the percentage of tumour cells with positive nuclear staining. A score of > 1% was considered positive [26]. HER2 was graded according to the ASCO/CAP HER2 testing guideline [27].

Based on expression of ER, PR, HER2 and Ki67, tumours were divided into four intrinsic molecular subtypes: Luminal-A, Luminal-B, HER2-positive and TNBC (basal-like), according to surrogate definitions of the ESMO guideline [28].

### Digital image analysis

**Fig. 2:**
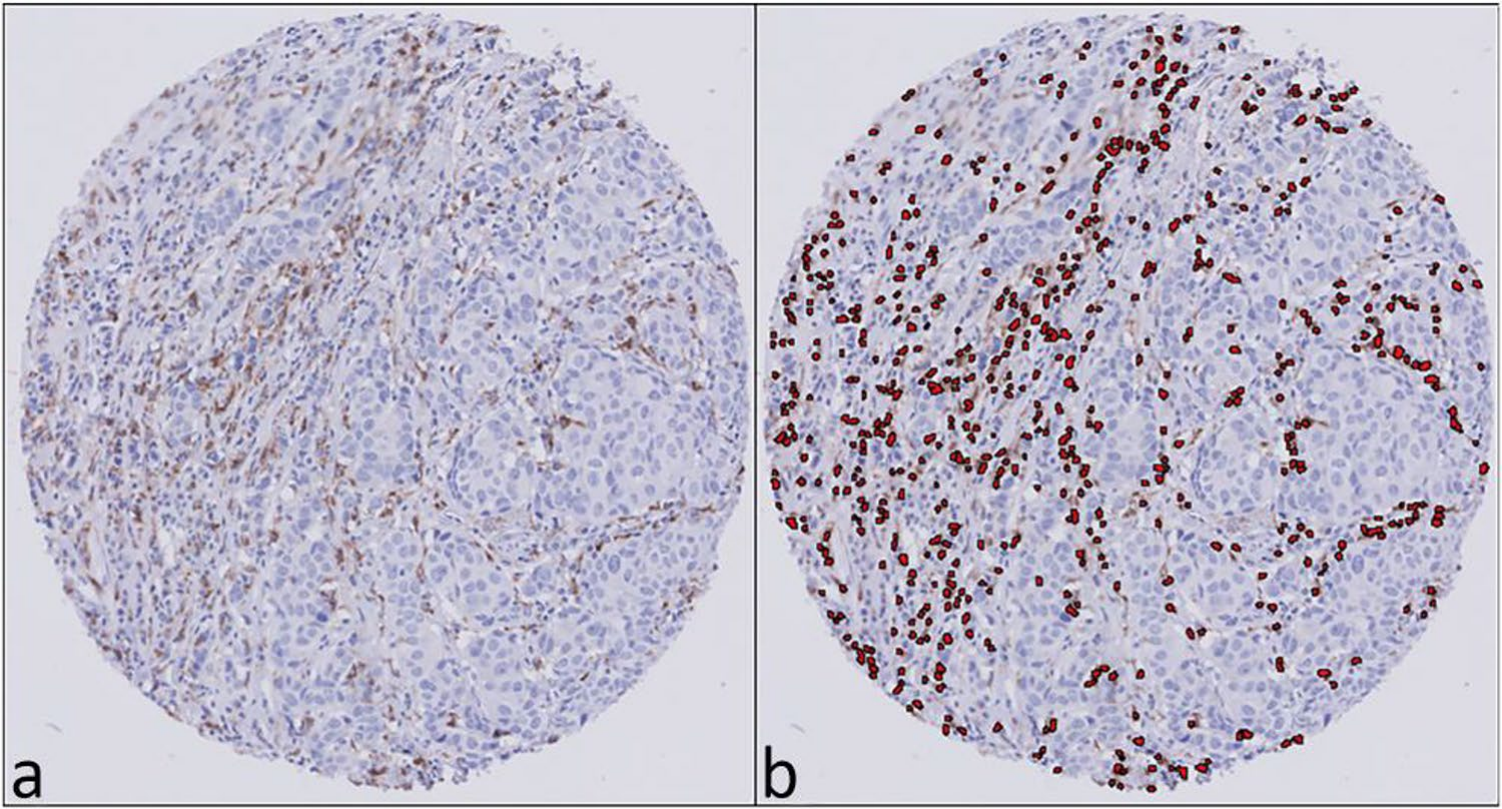
Digital image analysis of macrophage infiltration in representative TMA cores. Immunohistochemical staining for CD68 (**a**), digital image analysis of CD68-positive TAMs (**b**)

Digital images of the stained TMA slides were obtained by a Philips UltraFast Scanner (Philips, The Netherlands). The DIA platform used was Visiopharm Integrator System (VIS) version 7.0.1.318 (Visiopharm, Denmark). Ki67 was scored using a CE-IVD-approved DIA algorithm. An application-based algorithm was developed to detect the percentage and number of macrophages based on positive cytoplasmic staining for CD68, CD163, CSF-1R and CD206. The algorithm detects cells and classifies positive cells based on the size of the nuclei and the amount of surrounding staining (Fig. 2).

First, the TMAs were de-arrayed using the Tissue array module. A grid was superimposed on the digitalized TMA slides and manually and automatically adjusted to fit all cores. Next, the individual applications for the specific stains were run to detect and count the total number of macrophages and the number of M2-like macrophages per tumour core. An average score per tumour was determined and used to calculate the number of M1-like macrophages by subtracting the number of M2-like macrophages from the total number of macrophages. Additionally, the M2:M1 ratio was calculated.

### Definitions of terms

M2-like TAMs, detected by one of the M2-like macrophage markers, will be referred to as ^CD163^M2-like, ^CSF−1R^M2-like or ^CD206^M2-like TAMs. Similarly, M1-like macrophages, calculated by subtracting the number of M2-like TAMs detected by CD163, CSF-1R or CD206 from the total number of CD68 + macrophages, will be referred to as ^CD163^M1-like, ^CSF−1R^M1-like or ^CD206^M1-like TAMs. M2-like:M1-like TAM ratios will be described as ^CD163^ratio, ^CSF−1R^ratio or ^CD206^ratio.

### Statistical analysis

Statistical analyses were performed with IBM SPSS Statistics 25. Differences in TAM subsets between breast cancer subtypes were assessed with the Kruskal–Wallis test. Correlations between the different TAM markers were evaluated using Spearman’s correlation. Correlations between TAM subset counts and clinicopathological parameters were studied with Spearman’s correlation test for linear variables, a Mann–Whitney U test for binary variables and the Kruskal–Wallis test for categorical variables. Survival analyses were performed with univariate Cox regression analyses. Two-sided *P* values ≤ 0.05 were considered statistically significant. We used a Bonferroni correction to adjust for multiple testing when we studied correlations between TAM subsets and clinicopathological features, and when assessing the prognostic value of TAM subsets. In these cases, *P* values ≤ 0.005 were considered statistically significant.

## Results

### Patient characteristics

Clinicopathological characteristics are shown in Table 1. The median follow-up for the full cohort was 153 months (interquartile range [IQR] 100–174 months), 169 months (IQR 136–180 months) for the Luminal-A group, 140 months (IQR 110–171) for the Luminal-B group, 129 months (IQR 94–157 months) for the HER2-positive group and 109 months (IQR 48–152 months) for the TNBC group.

**Table 1:** Patient and tumour characteristics

*N* (%)				
	Luminal-A	Luminal-B	HER2-positive	TNBC
*Number of patients*	150 (43.2)	80 (23.1)	31 (8.9)	86 (24.8)
Age, years				
Median (range)	56.7 (35.0–92.2)	53.6 (31.3–80.7)	56.0 (30.3–74.2)	52.7 (26.6–90.1)
*Sex*				
Female	149 (99.3)	79 (98.8)	31 (100.0)	86 (100.0)
Male	1 (0.7)	1 (1.2)	0 (0.0)	0 (0.0)
*Histological type*				
Ductal (no special type)	124 (82.7)	75 (93.8)	27 (87.1)	72 (83.7)
Lobular	21 (14.0)	1 (1.2)	1 (3.2)	1 (1.2)
Other	5 (3.3)	4 (5)	3 (9.7)	13 (15.1)
*Histological grade*				
I	54 (36.0)	2 (2.4)	2 (6.5)	2 (2.3)
II	72 (48.0)	29 (36.3)	8 (25.8)	12 (14.0)
III	24 (16.0)	49 (61.3)	21 (67.7)	72 (83.7)
*ER status*				
Positive	150 (100.0)	77 (96.3)	0 (0.0)	0 (0.0)
Negative	0 (0.0)	3 (3.7)	31 (100.0)	86 (100.0)
*PR status*				
Positive	128 (85.3)	62 (77.5)	0 (0.0)	0 (0.0)
Negative	22 (14.7)	18 (22.5)	31 (100.0)	86 (100.0)
*HER2 immunohistochemistry*				
0	89 (59.3)	8 (10)	1 (3.2)	80 (93.0)
1 +	54 (36.0)	9 (11.2)	1 (3.2)	5 (5.8)
2 +	7 (4.7)	16 (20)	1 (3.2)	1 ()1.2
3 +	0 (0.0)	47 (58.8)	28 (90.3)	0 (0.0)
Tumour size (mm)				
Median (range)	13.5 (4.0–70.0)	18.5 (6.0–100.0)	20.0 (5.0–65.0)	19.0 (4.0–140.0)

*N*—number of patients, %—percentage, *mm—*millimetre

### TAM subset numbers vary across breast cancer subtypes

**Fig. 3:**
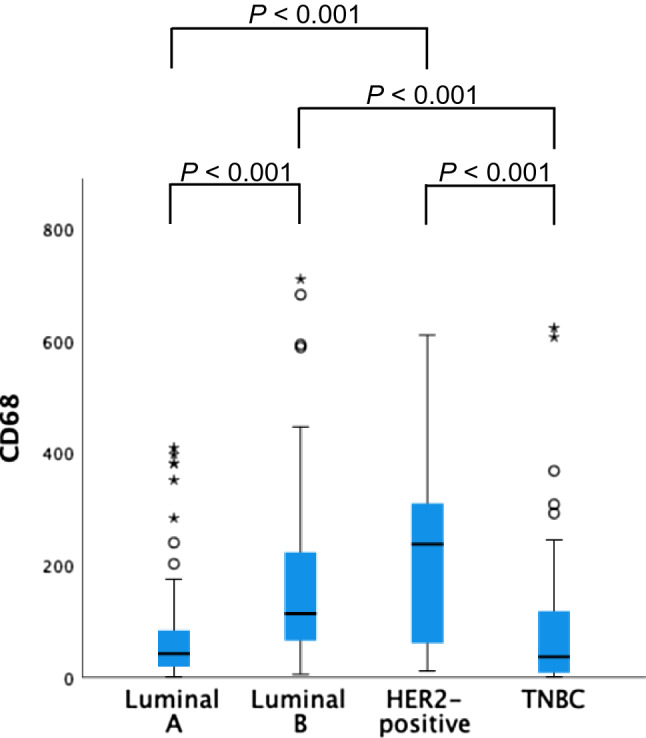
Distribution of CD68 + TAM numbers per breast cancer subtype. *P* values > 0.05 not shown

**Fig. 4:**
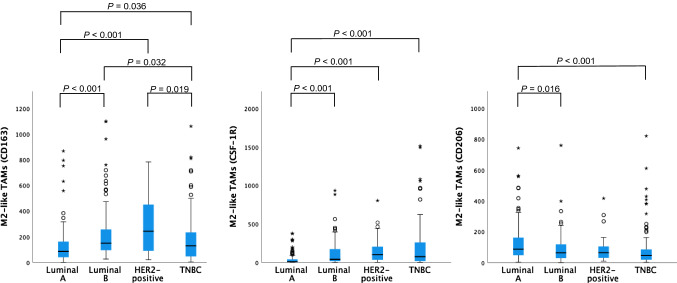
Distribution of M2-like TAM numbers per breast cancer subtype. *P* values > 0.05 not shown

**Fig. 5:**
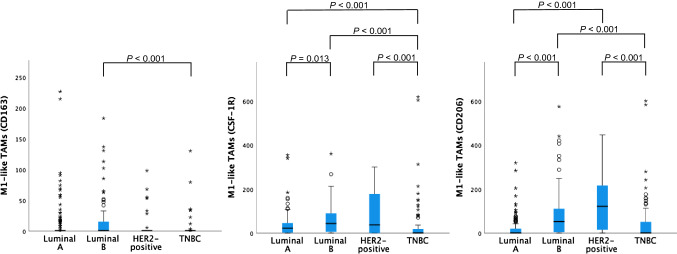
Distribution of M1-like TAM numbers per breast cancer subtype. *P* values > 0.05 not shown

^CD163^M2-like TAMs were more abundantly present in the HER2-positive tumours than in the Luminal-A or TNBC samples. ^CSF−1R^M2-like TAM numbers were highest in the TNBC and HER2-positive samples. In the Luminal-A group, ^CD206^M2-like TAM numbers were higher than in the Luminal-B and TNBC groups but did not differ from the HER2-positive group.

^CD163^M1-like TAM numbers were highest in the Luminal-B group. ^CSF−1R^M1-like and ^CD206^M1-like TAM numbers were higher in the HER2-positive group than in the Luminal-A and TNBC groups.

Distributions of TAM subset numbers per breast cancer subtype are shown in Figs. 3, 4 and 5. CD68 + TAM counts were higher in the HER2-positive group compared to the Luminal-A, Luminal-B and TNBC groups.

### CD68, CD163 and CSF-1R TAM numbers are strongly correlated with each other

CD68 + TAM numbers strongly correlated positively with ^CD163^M2-like TAM (*r* = 0.67, *P* < 0.001) and ^CSF−1R^M2-like TAM numbers (*r* = 0.47, *P* < 0.001), but only weakly with ^CD206^M2-like TAM numbers (*r* = 0.06, *P* = 0.260). ^CD163^M2-like TAMs and ^CSF−1R^M2-like TAMs (*r* = 0.50, *P* < 0.001) also correlated strongly. The numbers of ^CD206^M2-like TAMs and ^CD163^M2-like TAMs (*r* = 0.22, *P* < 0.001) or ^CSF−1R^M2-like TAMs (*r* = 0.22, *P* < 0.001) correlated weakly.

### Total number of CD68 + macrophages and M2-like TAM numbers positively correlate with unfavourable tumour characteristics in Luminal-B breast cancer

In the Luminal-B group, the number of CD68 + macrophages positively correlated with tumour grade (*P* < 0.001) (Table 2). Also, the number of ^CD163^M2-like and ^CSF−1R^M2-like macrophages (*P* < 0.001) and the ^ratio^CSF-1R (*P* = 0.001) were related to tumour grade.

**Table 2:** Correlations between TAM subset numbers and clinicopathological characteristics in Luminal-B breast cancer

Clinicopathological features		CD68	CD163			CSF-1R			CD206		
		Total	M2-like	M1-like	Ratio	M2-like	M1-like	Ratio	M2-like	M1-like	Ratio
Age^1^	*r*	− 0.207	− 0.237	− 0.032	− 0.134	− 0.281	0.128	− 0.242	− 0.009	− 0.115	0.071
	*P*	0.065	0.034	0.777	0.237	0.011	0.259	0.031	0.935	0.311	0.534
Menopausal status^2^	*P*	0.228	0.475	0.084	0.186	0.409	0.441	0.462	0.630	0.654	0.425
Premenopausal	Median	129.33	153	1	106	57.33	61.67	0.70	49.67	78.33	0.81
Perimenopausal	Median	62.67	191.67	1	191.67	28	32	1.83	75.67	15.5	3.26
Postmenopausal	Median	102.17	131.42	1	64	33.42	54.33	0.75	67.83	52.67	1.25
Tumour grade^2^	*P*	< 0.001*	< 0.001*	0.644	0.048	< 0.001*	0.439	0.001*	0.175	0.071	0.663
Grade 1	Median	50.17	102.33	1	102.33	27.5	22.67	4.88	37.5	12.67	5.56
Grade 2	Median	85	95.67	1	57	19	46.67	0.34	58	37.33	1.77
Grade 3	Median	136.33	216	1	191.67	102	39.5	1.87	75.67	83.67	0.80
Tumour size^1^	*r*	− 0.007	0.083	− 0.071	0.102	0.109	− 0.153	0.175	− 0.045	0.008	0.006
	*P*	0.948	0.462	0.532	0.367	0.334	0.177	0.121	0.693	0.944	0.955
Lymph node status^3^	*P*	0.650	0.760	0.712	0.813	0.661	0.472	0.953	0.622	0.788	0.937
Positive	Median	116.33	155	1	130.67	53.67	63.33	0.75	75.33	61.67	1.07
Negative	Median	109.33	132.5	1	104.83	34.44	38.17	0.75	57.5	46.67	0.88
Lymphovascular invasion^3^	*P*	0.981	0.880	0.641	0.664	0.683	0.674	0.739	0.709	0.775	0.647
Positive	Median	125.33	174.00	1	174.00	30.33	63.83	1.29	77.83	39.83	2.21
Negative	Median	110.58	132.5	1	101.5	42.33	40.42	0.75	58.33	52.17	0.87

*P ≤ 0.005

^1^Spearman’s rho

^2^Kruskal–Wallis test

^3^Mann–Whitney U test

r—correlation coefficient, *P—P* value, Median—median TAM subset number

### TAM subset numbers do not correlate with tumour characteristics in Luminal-A, HER2-positive and triple-negative breast cancer

In the Luminal-A, HER2-positive and TNBC groups, CD68 + pan-macrophage numbers did not correlate with any clinicopathological parameters (Tables 3, 4 and 5). Similarly, no correlations with clinicopathological parameters were found for M2-like or M1-like TAM numbers or with ratios of M2-like to M1-like TAMs for any of the markers.

**Table 3:** Correlations between TAM subset numbers and clinicopathological characteristics in Luminal-A breast cancer

Clinicopathological features		CD68	CD163			CSF-1R			CD206		
		Total	M2-like	M1-like	Ratio	M2-like	M1-like	Ratio	M2-like	M1-like	Ratio
Age^a^	*r *	− 0.176	− 0.153	0.006	− 0.105	− 0.134	− 0.096	− 0.015	0.074	− 0.128	0.139
	*P*	0.031	0.061	0.944	0.200	0.101	0.245	0.859	0.371	0.119	0.090
Menopausal status^b^	*P*	0.440	0.977	0.996	0.969	0.735	0.311	0.232	0.450	0.416	0.263
Premenopausal	Median	46.33	98.67	1	82	9.33	17.33	0.41	79.67	1	52
Perimenopausal	Median	56.5	94.7	1	97.4	8	34.92	0.19	65.83	1	43
Postmenopausal	Median	41	80	1	71.33	9.33	24.33	0.31	87.67	1	69.67
Tumour grade^b^	*P*	0.034	0.025	0.474	0.111	0.008	0.674	0.284	0.791	0.524	0.837
Grade 1	Median	32.5	70.83	1	67.5	6.67	16.17	0.35	87.67	1	66.17
Grade 2	Median	42.17	93.37	1 1	67.83	8	25.17	0.29	82.83	1	69
Grade 3	Median	66.5	150.75		150.75	22.67	30.33	0.43	90.17	1	76.17
Tumour size^a^	*r*	0.205	0.158	0.005	0.142	0.110	0.165	0.012	− 0.154	0.224	− 0.220
	*P*	0.012	0.054	0.953	0.083	0.180	0.044	0.887	0.060	0.006	0.007
Lymph node status^c^	*P*	0.353	0.291	0.958	0.336	0.510	0.229	0.797	0.578	0.718	0.561
Positive	Median	45.67	98.67	1	84.67	9.67	28.33	0.35	83.67	1	69.67
Negative	Median	39.17	81	1	65.33	7.5	17.83	0.31	93.67	1	69.5
Lymphovascular invasion^c^	*P*	0.975	0.117	0.214	0.075	0.417	0.749	0.367	0.094	0.266	0.109
Positive	Median	40.5	112.42	1	112.42	4	20.33	0.14	129.92	1	129.92
Negative	Median	41	76.67	1	68	8.33	21	0.35	82.67	1	69.33

*P  ≤  0.005

^a^Spearman’s rho

^b^Kruskal–Wallis test

^c^Mann–Whitney U test

*r*—correlation coefficient, *P—P* value, Median—median TAM subset number

**Table 4:** Correlations between TAM subset numbers and clinicopathological characteristics in HER2-positive breast cancer

Clinicopathological features		CD68	CD163			CSF-1R			CD206		
		Total	M2-like	M1-like	Ratio	M2-like	M1-like	Ratio	M2-like	M1-like	Ratio
Age^a^	*r*	− 0.069	0.080	0.175	0.040	0.081	− 0.272	0.233	− 0.110	0.104	0.060
	*P*	0.711	0.669	0.400	0.830	0.663	0.139	0.207	0.557	0.576	0.747
Menopausal status^b^	*P*	0.58	0.622	0.415	0.425	0.517	0.039	0.080	0.538	0.667	0.575
Premenopausal	Median	266.67	350.67	1	219	178.5	18	66.14	88.5	161.83	0.65
Perimenopausal	Median	222.33	210.83	1	148.5	115.42	7.33	3.24	51.17	78	0.85
Postmenopausal	Median	102	107.67	1	70.33	63	63.67	0.54	45	51.67	0.35
Tumour grade^b^	*P*	0.283	0.269	0.620	0.426	0.337	0.629	0.729	0.452	0.040	0.028
Grade 1	Median	51.33	76.17	17.17	59.68	46.5	21.17	33.81	39.17	17.17	22.68
Grade 2	Median	183.67	207.67	1	78.17	73.67	18.3	1.01	90.67	20.83	9.52
Grade 3	Median	237.33	300.67	1	243	125.67	3 45	2.55	64.67	170.33	0.36
Tumour size^a^	*r*	− 0.137	− 0.107	0.088	− 0.124	− 0.241	0.196	− 0.303	− 0.204 0.272	0.045	− 0.178
	*P*	0.462	0.567	0.638	0.507	0.191	0.291	0.098	0.098	0.809	0.339
Lymph node status^c^	*P*	0.453	0.105	0.726	0.175	0.058	0.689	0.206	0.264	0.922	0.626
Positive	Median	102	178.67	1	107.67	63	37.33	1.5	58	52.67	0.41
Negative	Median	251.83	367	1	367	165.42	38.17	2.79	67.17	144	0.62
Lymphovascular invasion^c^	*P*	0.518	0.788	0.542	0.829	0.162	0.353	0.196	0.389	0.808	0.914
Positive	Median	86.5	143.17	1	143.17	70.67	49	1.04	49.5	40.5	0.82
Negative	Median	237.33	243	1	118.3	125.67	22.33	2.55	64.67	122	0.57

*P ≤ 0.005

^a^Spearman’s rho

^b^Kruskal–Wallis test

^c^Mann–Whitney U test

*r*—correlation coefficient, *P—P* value, Median—median TAM subset number

**Table 5:** Correlations between TAM subset numbers and clinicopathological characteristics in TNBC

Clinicopathological features		CD68	CD163			CSF-1R			CD206		
		Total	M2-like	M1-like	Ratio	M2-like	M1-like	Ratio	M2-like	M1-like	Ratio
Age^a^	*R*	0.097	0.001	0.052	0.005	0.121	− 0.168	0.136	0.065	0.040	− 0.011
	*P*	0.374	0.989	0.633	0.967	0.267	0.123	0.212	0.555	0.712	0.919
Menopausal status^b^	*P*	0.734	0.075	0.040	0.015	0.730	0.249	0.505	0.622	0.821	0.856
Premenopausal	Median	35.5	175.83	1	131.33	49.17	1	19.17	48.5	1	18.3
Perimenopausal	Median	15.33	44	1	39.67	62.33	1	55	19.33	5.33	1.88
Postmenopausal	Median	37.33	142	1	142	82.67	1	82.67	61	1	20.67
Tumour grade^b^	*P*	0.055	0.034	0.181	0.037	0. 971	0.547	0. 859	0.446	0.270	0.929
Grade 1	Median	2.33	16.5	1	16.50	203.00	1	203.00	35.00	1	35.00
Grade 2	Median	14.67	61	1	46.33	63.00	1	36.00	36.67	1	25.00
Grade 3	Median	39.92	146.83	1	128.50	79.5	1	53.17	50.00	1	16.26
Tumour size^a^	*r*	− 0.008	1460.040	0.078	0.078	0.127	− 0.113	0.148	0.057	− 0.002	0.095
	*P*	0.946	0.719	0.479	0.480	0.248	0.305	0.262	0.605	0.986	0.388
Lymph node status^b^	*P*	0.877	0.779	0.349	0.493	0.727	0.625	0.810	0.412	0.931	0.666
Positive	Median	36	142	1	115	51.33	1	35.67	40.67	1	20.67
Negative	Median	35	112.67	1	89	97.33	1	62.33	48.33	1	17
Lymphovascular invasion^b^	*P*	0.141	0.028	0.208	0.023	0.767	0.021	0.109	0.981	0.083	0.136
Positive	Median	92.67	184	1	182	43.67	18.67	1.27	45.33	28.33	0.98
Negative	Median	24.33	94.17	1	81.67	75.83	1	58.67	45.5	1	20.67

*P ≤ 0.005

^a^Spearman’s rho

^b^Kruskal–Wallis test

^c^Mann–Whitney U test

*r*—correlation coefficient, *P—P* value, Median—median TAM subset number

### TAM subset numbers are not associated with disease outcome

Univariate survival analyses of the total study cohort did not show associations between TAM subset numbers and DFS or OS (Supplementary Table 1). Similarly, no associations between TAM subset counts and DFS or OS were found in patients with Luminal-A, Luminal-B, HER2-positive or triple-negative breast cancer (Supplementary Tables 2–5).

## Discussion

In this study, we found positive associations between high CD68 + TAM/^CD163^M2-like TAM numbers and higher tumour grade in the Luminal-B group. Furthermore, ^CSF−1R^M2-like TAMs and ^ratio^CSF-1R were related to high tumour grade in the Luminal-B group.

To our knowledge, we are the first who compared CD68, CD163, CSF-1R and CD206 for TAM detection and assessed their relation with clinicopathological characteristics in a large well-characterized series of intrinsic breast cancer subtypes. Furthermore, the long-term follow-up of the patients (median 153 months) allowed for adequate assessment of the relation between TAMs and patient outcome.

A meta-analysis of 16 studies assessing the relation of CD68 + , CD163 + and/or CD206 + TAMs with survival in primary, adjuvant- and neoadjuvant-treated patients with breast cancer, found that CD68 was a better outcome predictor than CD163 and CD206 [29]. However, none of the included studies compared all three markers in one breast cancer set. At the same time, it is increasingly recognized that the tumour microenvironment contains many inflammatory cell types that may contribute to tumour behaviour prognosis in a contradicting manner [6]. In addition, while early clinical trials with CSF-1R targeting compounds are being conducted, studies on CD68 + or CD163 + macrophages as a therapeutic target are lacking [30–34]. Therefore, a direct comparison of the clinical relevance of these three M2-like TAM markers in breast cancers has not yet been performed, but may be important, as this may provide a rationale for selective macrophage subset targeting in patients with breast cancer.

We hypothesized that CD163, CSF-1R and CD206 are specific M2-like macrophage markers that would provide accurate and comparable M2-like TAM numbers. However, the TAM numbers detected by the M2-like markers in our study varied considerably between and within breast cancer subtypes and did not identify a similar M2-like subset. We did find a moderate correlation between CD68, CD163 and CSF-1R, but only a weak correlation with CD206. Furthermore, in some cases, the number of ^CD163^M2-like TAMs exceeded the CD68 + total macrophage number. These findings indicate a non-specificity of these markers for macrophages. Moreover, macrophages are highly plastic and exhibit functional and phenotypical diversity, depending on environmental stimuli [35, 36]. It seems likely that macrophages play multifunctional roles in development and progression of breast cancer. This functional heterogeneity is reflected by a heterogeneous expression of TAM markers [37–40]. Therefore, selectively identifying specific TAM subsets remains difficult and may complicate TAM-targeted therapy [35]. Immunohistochemical staining for multiple TAM markers, for example double or triple staining, may overcome this difficulty.

In contrast to previous studies, neither total macrophages nor TAM subsets correlated with DFS or OS for the whole group or per breast cancer subtype. Others reported that high expression of CD68, CD163 and CD206 was predictive of poor OS, breast cancer-specific survival, or recurrence-free survival [23, 41–45]. These studies were smaller than our study, comprising between 107 and 278 patients, but showed relatively high recurrence and death rates [23, 41–43]. Selection bias might therefore have played a role in the results of these studies. One large study of 562 patients with breast cancer in which TAMs were expressed as the number of positive cells per TMA core with DIA is most comparable to our study. This study did not find an association between CD68 + and CD163 + TAMs and survival [24].

This study has strengths and limitations. Strengths of our study are the large breast cancer series with a long-term follow-up and availability of well-characterized clinical data. Furthermore, instead of manual counting we used DIA to objectively quantify TAM numbers in a standardized manner. Limitations are the small breast cancer subtype groups and the fact that other immune microenvironment markers, e.g. tumour-infiltrating lymphocytes, were not considered. Concerning the M1-like TAM subset, we preferably would have detected the M1-like TAM subset using a specific marker. Unfortunately, such a marker is currently not available [46].

In conclusion, we found that total TAMs and M2-like TAM subsets and their ratios were related to our series’ clinicopathological characteristics in Luminal-B breast cancer. Therefore, based on these data we suggest that it is important to consider TAM subsets and their ratio, per specific breast cancer subtype. Prospective series are needed to identify the clinically most relevant marker for M2-like macrophages and other (immune) environment markers. Ultimately, this may support rational macrophage targeting in breast cancer.

## Supplementary Information

Below is the link to the electronic supplementary material.

Supplementary file1 (PDF 223 kb)

## Data Availability

The datasets generated and/or analysed during the current study are available from the corresponding author on reasonable request.
