# Supplementary material for: Assessing the role of tumour-associated macrophage subsets in breast cancer subtypes using digital image analysis
Source: Breast Cancer Res Treat. 2023 Jan 9;198(1):11–22. doi: 10.1007/s10549-022-06859-y (PMC9883348; doi:10.1007/s10549-022-06859-y)
Supplement: Supplementary file 1 — Supplementary file1 (PDF 223 kb) [file 10549_2022_6859_MOESM1_ESM.pdf]

Assessing the role of tumour-associated macrophage subsets in breast cancer subtypes using digital image analysis

Breast Cancer Research and Treatment

Mieke C Zwager, Rico Bense, Stijn Waaijer, Si-Qi Qiu, Hetty Timmer-Bosscha, Elisabeth GE de Vries, Carolien P Schröder, Bert van der Vegt

Corresponding author

B. van der Vegt, MD, PhD,

University of Groningen, University Medical Center Groningen

Department of Pathology and Medical Biology

PO Box 30001, 9700 RB Groningen.

Telephone: +31503615272

E-mail: b.van.der.vegt@umcg.nl

Supplementary data

Supplementary Table 1. Univariate analysis of DFS and OS of the total study cohort

	DFS			OS		
	**HR**	**95% CI**	***P***	**HR**	**95% CI**	***P***
**CD68**	0.999	0.997-1.001	0.162	0.998	0.996-1.000	0.067
**^{CD163}M2-like**	0.999	0.998-1.001	0.304	0.999	0.998-1.000	0.160
**^{CD163}M1-like**	0.997	0.989-1.004	0.360	0.995	0.986-1.004	0.288
**^{CD163}ratio**	1.000	0.999-1.001	0.624	0.999	0.998-1.001	0.380
**$^{CSF\text{-}1R}$M2-like**	1.000	0.999-1.001	0.774	1.000	0.999-1.001	0.649
**$^{CSF\text{-}1R}$M1-like**	0.999	0.996-1.001	0.295	0.997	0.993-1.000	0.084
**$^{CSF\text{-}1R}$ratio**	1.000	0.999-1.001	0.747	1.000	0.999-1.001	0.519
**^{CD206}M2-like**	1.001	0.999-1.002	0.501	1.000	0.998-1.002	0.763
**^{CD206}M1-like**	0.998	0.996-1.001	0.185	0.998	0.995-1.001	0.121
**^{CD206}ratio**	1.001	0.999-1.002	0.334	1.001	0.999-1.002	0.335

HR, hazard ratio; 95% CI, 95% confidence interval; *P*, *P*-value.

Supplementary Table 2. Univariate analysis of DFS and OS in Luminal-A breast cancer

	DFS			OS		
	**HR**	**95% CI**	***P***	**HR**	**95% CI**	***P***
**CD68**	1.000	0.997-1.003	0.922	0.999	0.995-1.003	0.693
**^{CD163}M2-like**	1.000	0.998-1.002	0.715	0.999	0.996-1.001	0.392
**^{CD163}M1-like**	0.995	0.984-1.005	0.330	0.994	0.981-1.008	0.407
**^{CD163}ratio**	1.000	0.998-1.002	0.830	0.999	0.997-1.002	0.555
**$^{CSF\text{-}1R}$M2-like**	0.999	0.995-1.003	0.507	0.996	0.989-1.002	0.158
**$^{CSF\text{-}1R}$M1-like**	1.000	0.996-1.004	0.966	1.000	0.996-1.005	0.860
**$^{CSF\text{-}1R}$ratio**	0.998	0.994-1.003	0.482	0.996	0.989-1.003	0.241
**^{CD206}M2-like**	1.000	0.998-1.003	0.767	1.000	0.997-1.002	0.756
**^{CD206}M1-like**	1.000	0.995-1.005	0.945	0.999	0.993-1.005	0.761
**^{CD206}ratio**	1.001	0.999-1.003	0.573	1.000	0.998-1.002	0.895

HR, hazard ratio; 95% CI, 95% confidence interval; *P*, *P*-value.

Supplementary Table 3. Univariate analysis of DFS and OS in Luminal-B breast cancer

	DFS			OS		
	**HR**	**95% CI**	***P***	**HR**	**95% CI**	***P***
**CD68**	0.999	0.995-1.003	0.709	0.998	0.994-1.004	0.744
**^{CD163}M2-like**	0.998	0.995-1.001	0.294	0.999	0.995-1.002	0.437
**^{CD163}M1-like**	1.003	0.991-1.015	0.623	1.000	0.984-1.017	0.980
**^{CD163}ratio**	0.998	0.996-1.001	0.308	0.999	0.995-1.002	0.460
**$^{CSF\text{-}1R}$M2-like**	0.999	0.997-1.002	0.676	1.000	0.997-1.003	0.955
**$^{CSF\text{-}1R}$M1-like**	0.997	0.989-1.006	0.497	0.997	0.986-1.007	0.530
**$^{CSF\text{-}1R}$ratio**	0.998	0.993-1.002	0.327	0.999	0.995-1.003	0.679
**^{CD206}M2-like**	1.000	0.995-1.005	0.980	1.000	0.993-1.007	0.811
**^{CD206}M1-like**	0.999	0.994-1.004	0.749	0.998	0.992-1.004	0.549
**^{CD206}ratio**	0.998	0.992-1.005	0.642	0.998	0.990-1.007	0.700

HR, hazard ratio; 95% CI, 95% confidence interval; *P*, *P*-value.

Supplementary Table 4. Univariate analysis of DFS and OS in HER2-positive breast cancer

	DFS			OS		
	**HR**	**95% CI**	***P***	**HR**	**95% CI**	***P***
**CD68**	0.999	0.994-1.004	0.697	0.999	0.994-1.004	0.787
**^{CD163}M2-like**	1.000	0.997-1.004	0.834	1.001	0.998-1.005	0.462
**^{CD163}M1-like**	0.973	0.921-1.027	0.322	0.938	0.798-1.103	0.438
**^{CD163}ratio**	1.001	0.998-1.004	0.483	1.002	0.999-1.005	0.261
**$^{CSF-1R}$M2-like**	0.999	0.995-1.003	0.599	1.000	0.996-1.004	0.957
**$^{CSF-1R}$M1-like**	0.999	0.992-1.006	0.816	0.995	0.985-1.005	0.367
**$^{CSF-1R}$ratio**	0.998	0.994-1.003	0.477	0.999	0.995-1.003	0.738
**^{CD206}M2-like**	0.996	0.986-1.007	0.494	0.996	0.985-1.008	0.550
**^{CD206}M1-like**	1.001	0.995-1.006	0.792	1.001	0.995-1.007	0.723
**^{CD206}ratio**	1.002	0.991-1.013	0.746	1.002	0.991-1.014	0.666

HR, hazard ratio; 95% CI, 95% confidence interval; *P*, *P*-value.

Supplementary Table 5. Univariate analysis of DFS and OS in TNBC

	DFS			OS		
	**HR**	**95% CI**	***P***	**HR**	**95% CI**	***P***
**CD68**	0.999	0.995-1.003	0.562	0.997	0.993-1.002	0.248
^{CD163}**M2-like**	1.000	0.999-1.002	0.690	0.999	0.997-1.001	0.418
^{CD163}**M1-like**	1.005	0.989-1.023	0.526	1.008	0.991-1.025	0.378
^{CD163}**ratio**	1.001	0.999-1.002	0.523	0.999	0.997-1.001	0.499
$^{CSF\text{-}1R}$**M2-like**	1.000	0.999-1.001	0.752	1.000	0.999-1.001	0.566
$^{CSF\text{-}1R}$**M1-like**	0.999	0.994-1.003	0.575	0.994	0.985-1.004	0.247
$^{CSF\text{-}1R}$**ratio**	1.000	0.999-1.001	0.688	1.000	0.999-1.001	0.496
^{CD206}**M2-like**	1.001	0.998-1.003	0.540	1.001	0.998-1.003	0.516
^{CD206}**M1-like**	0.998	0.993-1.003	0.393	0.995	0.988-1.003	0.218
^{CD206}**ratio**	1.000	0.998-1.003	0.681	1.001	0.999-1.003	0.488

HR, hazard ratio; 95% CI, 95% confidence interval; *P*, *P*-value.
